# The effects of playing Nintendo Wii on depression, sense of belonging and social support in Australian aged care residents: a protocol study of a mixed methods intervention trial

**DOI:** 10.1186/s12877-015-0107-z

**Published:** 2015-09-03

**Authors:** Jessica Chesler, Suzanne McLaren, Britt Klein, Shaun Watson

**Affiliations:** School of Health Sciences and Psychology, Faculty of Health, Federation University Australia, PO Box 663, Ballarat, VIC 3353 Australia; DVC-R&I Portfolio & Collaborative Research Network, Federation University Australia, Ballarat, Australia; National Institute for Mental Health Research, The Australian National University, Canberra, Australia

## Abstract

**Background:**

The proportion of people aged 65 or older is the fastest growing age group worldwide. Older adults in aged care facilities have higher levels of depression, and lower levels of social support and sense of belonging compared with older adults living in the community. Research has begun to assess the effectiveness of interventions to improve the mental health of residents and has found both cognitive and physical benefits of video game playing. The benefits of playing these games in a group may also lead to greater social interaction and decreased loneliness. The current study aims to investigate an intervention program designed to foster relationships among older adults in care based on shared interests. Residents will be assessed on the effectiveness of a 6 week program of playing Wii bowling in comparison to a control group.

**Method/Design:**

Participants will be allocated to the intervention (Wii bowling) or the control group based on their place of residence. Participants in the intervention group will be invited to participate in Wii bowling twice weekly, with up to three other residents for a period of 6 weeks. Residents in both conditions will be assessed for depression, social support, sense of belonging, and current self-rated mood at pre-intervention (0 weeks), post-intervention (6 weeks), and at 2-month follow up (14 weeks). Qualitative data on social interaction between group members will also be collected at weeks 1, 3, and 6. Both groups will receive a Wii console after week 6 to establish if residents and staff engage with the Wii without intervention.

**Discussion:**

The Wii provides a user friendly platform for older adults to use video games, and it incorporates both social and competitive aspects in the game play. Existing research has not extensively investigated the social aspects of using this type of technology with older adults. If found to be effective, incorporating Wii games into an activity schedule may benefit the mental health of older adults living in care by establishing an intervention that is fun, economical, and easy to use.

**Trial Registry:**

Australian New Zealand Clinical Trials Registry: ACTRN12614000445673

## Background

The World Health Organisation has estimated that the global population of older adults will grow by 223% by the year 2050 [1]. In 2050 it is estimated that there will be 2 billion older adults, of which 80% are expected to be living in developing countries [1]. Due to the increasing time spent in old age and the high level of disability in older adults, there is an increasing need for residential aged care facilities, community care, and flexible care services [2].

Older adults in residential care are at an increased risk of a number of mental health disorders when compared to community samples. Research indicates that 40.5% of residents in high level care and 25.4% of residents in low level care experience depression [3]. Older adults are also at risk of experiencing loneliness, with approximately 10% of older adults in care reporting frequent loneliness [4]. Harper [5] has found that the aged care environment does not promote well-being or the formation of meaningful friendships even though residents are often with other people. Loneliness in older adults is strongly associated with higher levels of depression, even after controlling for variables such as gender, age, ethnicity, education, income, marital status, social support, and perceived stress [6, 7]. Older adults in residential care are at an increased risk of a number of mental health disorders even though residents are in an environment that has been designed to provide social support, alleviate boredom, and decrease loneliness along with providing medical support. Knight and Mellor [8] have proposed that a combination of unfulfilling social activities and constant interactions with unfamiliar people can lead to the development of poor mental health in aged care residents.

Low levels of sense of belonging have been implicated in the poor mental health of older adults who live in aged care facilities [9]. Recent research has shown that sense of belonging partially mediates the relationship between place of residence and depression in older adults, with living in assisted living facilities being associated with lower levels of belonging [9]. Lower levels of belonging is, in turn, associated with higher levels of depressive symptoms [9]. Other research has indicated that residents feel that they do not fit in with those around them, despite aged care providers believing that the provision of a range of social activities facilitates a sense of belonging among residents [8]. The above research highlights the importance of sense of belonging to the well-being of aged care residents and emphasises the need for interventions to enhance a sense of belonging within the aged care environment. Increasing the levels of sense of belonging should be associated with an increase in the overall psychological health and wellbeing of residents [10].

### Gaming

Video games can provide older adults with a number of physical and psychological benefits. Video games are not just used for fun and entertainment. The term “serious gaming” has been used to identify games used for purposes such as education, training, advertising, research, and health promotion [11]. Recently, games have been developed that also incorporate the use of the whole body. These games have been termed “exergames” as they can improve health, physical fitness, and coordination through the combination of physical activity and game play [11]. A systematic review of the physical and cognitive effects of older adults playing physically interactive computer games was conducted [12]. Across 12 studies it was found that interactive computer games were safe, and are an effective way to increase physical activity in older adults. The use of the computer games was also associated with improvements in a range of physical and cognitive outcomes, including self-esteem [13], confidence [14] and balance [15].

A popular game console that is used with older adults is the Nintendo Wii. The Wii is able to track spatial movements and incorporate this into game play [16]. The cost effectiveness of commercial systems such as the Wii make this a feasible option for aged care facilities [17]. Marston [18] found that the Wii console was easier for older adults to use when compared to other more traditional digital games.

The Wii has been shown to be of benefit to older adults who are suffering from sub-syndromal depression. Rosenberg et al. [19] conducted a pilot study with community living older adults with sub-syndromal depression. At the end of three months, participant’s levels of depression decreased and there was an increase in quality of life. Kahlbaugh, Sperandio, Carlson, and Hauselt [20] investigated the effects of playing the Wii on physical activity, loneliness, and mood with older adults in a residential facility. Residents were asked to either play Wii games of their choice or watch television with an undergraduate student. It was found that the group playing Wii had a decrease in loneliness whereas the group watching television had an increase in loneliness by the end of week 10. There was no overall change in negative mood or physical activity levels over the 10 weeks, however during each week there was a non-significant increase in reported positive mood [20].

Playing Wii has also been found to improve the physical activity levels and psychological quality of life when compared to a control group [21]. Residents were asked to play a variety of Wii sports games over 8 weeks. Analyses of the group interview data indicated that residents found the games fun and provided an avenue for greater socialization. By week 8, residents' overall quality of life had improved in the domains of psychological and physical health [21]. In another study using the Wii, participants were divided into three groups, playing Wii with others, playing Wii alone, and a control group who played board games [22]. At the conclusion of the study, the residents in the Wii conditions scored significantly higher on self-esteem, physical activity, and positive affect, and significantly lower on loneliness when compared to the control group. No difference was found between the groups playing Wii alone and with others [22].

No previous study has systematically measured the effect of using the Wii on depression, sense of belonging, or social support. Social interaction has been measured previously at the conclusion of the intervention, but not throughout the intervention [21]. Depression is a significant issue for residents' well-being, along with a lack of social support and belonging within care facilities. Playing the Wii console is a potential intervention to increase the overall psychological health of residents. Further, no study has utilised a control group to test the use of the Wii console in care facilities measuring depression, social support, and a sense of belonging as the outcome measures.

### Study aims

The aim of the current research is to investigate the mental health of aged care residents (aged 65 years or older) who are encouraged to participant in a group activity involving the Wii console. Participants will be allocated to either an intervention group that involves playing Wii bowling with other residents or to a control group who do not participate. The primary aim of the study is to examine the effectiveness of the Wii to decrease the level of depressive symptoms and increase a sense of belonging, increase self-reported mood, and increase social support at post-intervention and 2-month follow up. A secondary aim of the intervention is to increase the level of social interaction between participants.

## Methods/Design

### Study design

**Fig. 1:**
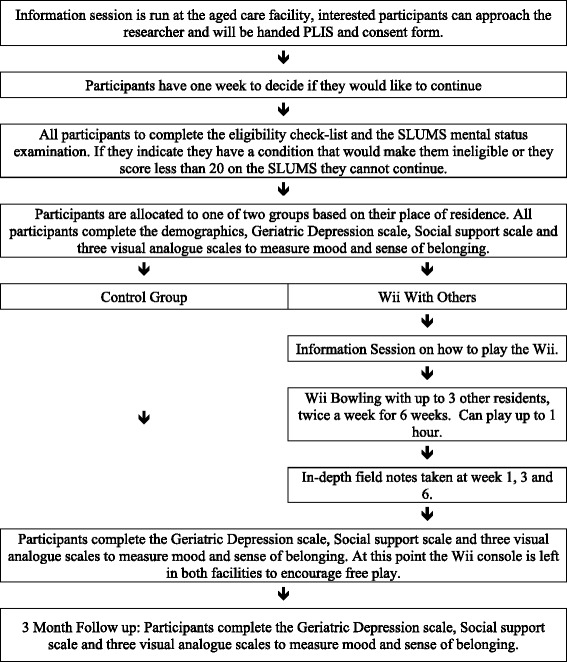
Flow chart of study design

This trial will utilise a mixed-methods, quasi-experimental trial design where an active Wii intervention will be compared to a control (wait condition) utilising aged care facility residents. Residents allocated to the Wii intervention condition will be asked to play Wii bowling for 6 weeks. Those residents in aged care facilities allocated to the control condition will not have access to the Wii intervention during the first 6 weeks. The intervention group will be compared to residents in an aged care facility  with no intervention. All participants meeting inclusion criteria will be assessed using paper and pen surveys at pre-intervention (week 0), post-intervention (week 6), and 2-month follow up (week 14). Qualitative interval analysis will also be conducted throughout the intervention (weeks 1, 3 and 6). The aged care facilities for both the control and intervention group will be provided with the Wii and instructions for use to encourage free play after week 6 (Fig. 1, Flow chart of study design). Recruitment began on 25 November 2013 and continued until November 2014.

### Participants

Participants will be recruited from their place of residence with the approval of the aged care facility manager. Four aged care facilities will be sought for participation. Exclusion criteria are: severe cognitive impairment or dementia as assessed by the Saint Louis Mental Status Examination (SLUMS, [23, 24]), diagnosis of bipolar affective disorder, schizophrenia, intellectual disability, severe hearing impairment, acute illness, inability to communicate in English due to non-English speaking background, age of less than 65 years, epilepsy or seizures, pacemaker or implanted medical device or physical disability preventing use of the Wii console. For this research, participants will be excluded if they received a score of 20 or less on the SLUMS exam indicating the presence of dementia or severe cognitive impairment [24]. These exclusion criteria are based on previous research and on the safety precautions provided in the instruction manual from the Wii. Written consent will be gained from both the participants and from the aged care facility manager.

### Intervention

The program consists of the residents playing Wii bowling in a group with up to three other residents for a period of 6 weeks, for up to 1 hour per session. Wii bowling has been selected as research shows that with older populations it is most popular Wii game and the easiest to learn [17] over the relatively short trial period. It is expected that residents will be able to easily master the game play to allow for social interaction to occur between residents.

Each group requires a minimum of two residents to run each week. The Wii will be located in a quiet area of the facility, and residents will play seated in a semi-circle to reduce the risk of falls. The participants will also be involved in an onsite training session run by the researcher before the commencement of the intervention. Residents will be advised that the purpose of the intervention is to learn a fun, new activity with others.

### Control group

The control group participants will be located in two different aged care facilities. The control group will not take part in any activities involving the Wii console in the first 6 weeks. The control and intervention group will be provided with a Wii console and staff training on how to play Wii bowling at the end of the 6 week intervention period.

### Administration of assessments

A researcher will attend each aged care facility that agrees to participate. A presentation will be given to outline the research, and what it would involve for the residents. One week later, a researcher will approach interested residents on site. Residents will be assessed on their levels of depression, social support, sense of belonging, and self-reported mood at pre-intervention (week 0), post-intervention (week 6), and 2-month follow up (week 14).

### Primary outcomes

The primary outcomes to be assessed are reduction in depressive symptoms and improvements in self-reported mood, sense of belonging, and levels of social support.

#### Depression

The Geriatric Depression Scale Short Form [24] will be used to measure depression. The scale utilises a simple *yes/no* format for ease of administration and consists of 15 items (e.g., Do you often feel helpless?). Higher scores indicate higher levels of depressive symptomatology in an individual. A cut off score of 7 or more will be used for this research with α = .91 [25]. The shorter version of the scale is able to differentiate depression from non-depressed older adults, and has a significant correlation with the original 30 item scale (*r* = .84, *p <* .001, [26]).

#### Social support

Social support will be measured using the Social Provisions Scale [27]. The participants will indicate on a 4-point scale the extent to which each statement describes their current level of social support (e.g., There are people who enjoy the same social activities I do). Responses range from strongly disagree to strongly agree. Cutrona and Russell [27] report coefficient alphas ranging between .65 to .70 and test-retest reliability coefficients ranging from .37 to .66 over a 2 week period.

#### Sense of belonging

To measure sense of belonging, a visual analogue scale will be utilised. The Sense of Belonging Visual Analogue Scale was developed to enable the 18-item Sense of Belonging Instrument [28] to be reduced to a two item visual analogue scale for ease of use. The Sense of Belonging Visual Analogue Scale measures the two theoretical concepts underlying the Sense of Belonging Instrument, these concepts are how much someone feels valued, and their fit with the surrounding environment. The Sense of Belonging Visual Analogue Scale presents participants with two 10 cm lines. At each end of the line opposing statements are placed asking residents about feeling valued and feeling as though they fit in. Respondents will be asked to place a vertical line indicating the extent to which they endorsed the concepts on either extreme. The placement of the line will be measured in centimetres, and rounded to the closest centimetre. Higher scores indicate greater levels of each concept.

The validity of the scale has been established by Morris [29], scores have been correlated with scores obtained on the psychological state sub-scale of the Sense of Belonging Instrument [28]. There was a significant, strong positive correlation between the two instruments (*rho* = .67, *p* < .001).

#### Mood

**Fig. 2:**
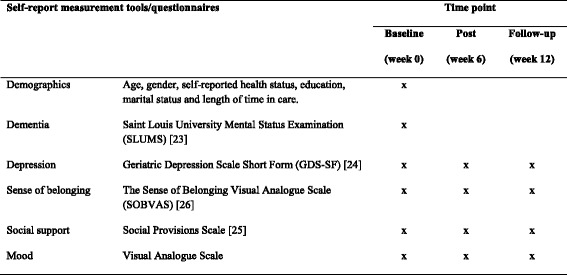
Instruments used for data collection

**Fig. 3:**
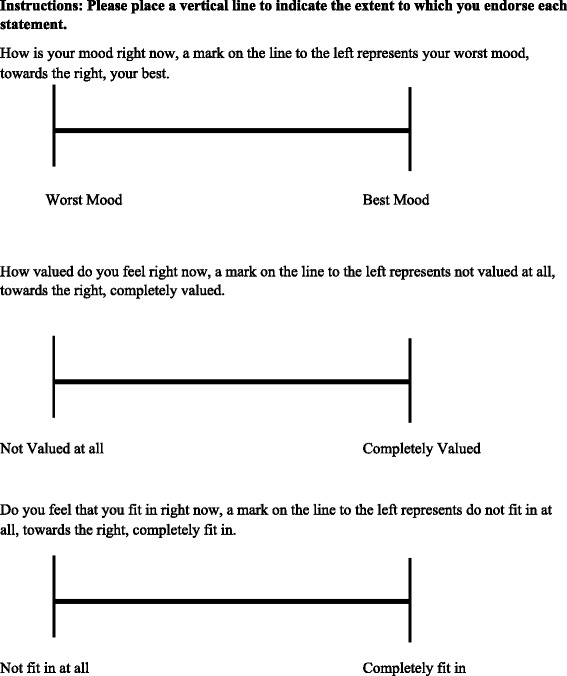
Visual analogue scales

To measure participant's current mood at the time of completing the questionnaire packages, participants will be asked to indicate “How is your mood right now?” on a visual analogue scale. The scale will be in the same format and scored in the same manner as the sense of belonging visual analogue scale. The residents will be presented with opposing statements at each end of a 10 cm line. The extreme positions will have the statements “worst mood” and “best mood”. Higher scores indicate a better mood. Currently, there is no validity for data for the mood scale (Fig. 2, Instruments used for data collection, Fig. 3, Visual analogue scales).

### Secondary outcomes

Participants in the intervention group playing Wii with others will also be involved in a qualitative interval interaction analysis. Onsite observational data will be collected during video game playing sessions in week 1, 3, and 6. Verbatim written transcripts will be made of these sessions. The observational data collected will focus on the interaction between group members to ascertain the type of social interactions that occurs, and if there is an increase over the course of the intervention period. Interaction data will be coded by the key researcher and then analysed utilising Nvivo 10. A researcher will be on site during each session to address any technical issues, but will not participate or interact with group members. Length of time spent playing, high scores, and attendance will be recorded by the researcher for each session. This data will be analysed to establish any differences between the two intervention facilities in game play time, and also to establish how long each resident is playing the game. Game play will be defined as when the resident is actively playing, or watching others play the game.

Post-intervention evaluation will also be conducted using a short answer questionnaire designed specifically for this research to enable participants to provide information about their experience of the intervention. This questionnaire will ask residents questions such as “What did you enjoy about playing the Wii”, “What didn’t you enjoy about playing the Wii”, “Would you prefer to play alone or with other people?” and “Would you continue to play the Wii in the future if it was available?”. These questions were chosen by the researcher to encourage the residents to express any positive or negative aspects of playing the game, as well as any limitations or difficulties they experienced. Staff opinions on the feasibility, ease of use, and enjoyment of the Wii console will also be obtained at the end of the 6 week intervention. Staff opinions will be obtained through semi-structured interviews following a similar format to the post-intervention evaluation questionnaire. This is to establish the staff response to the game play. To establish if residents are able to play Wii bowling without significant staff intervention in the activity, both groups (control and Wii) will be provided with the Wii console, games, and training on how to play at the end of week 6.

### Hypotheses

It is hypothesised that participants in the active Wii condition will demonstrate greater improvements in mood, depression, social support, and sense of belonging, in comparison to those in the control condition. Those in the active Wii condition are hypothesised to also maintain their improvements at the 2 month follow-up. In addition, it is hypothesised that those participants in the active Wii intervention will demonstrate higher levels of within group, positive group interactions from pre to post-intervention.

### Statistical analysis

To establish if there are any significant differences between the intervention and the control group on levels of dementia (SLUMS exam), residents' age [30], and length of time in care [31], between groups *t*-tests will be conducted. A series of chi-square tests will be conducted to establish if there are differences between groups and the demographic variables of relationship status, level of education, self-rated health status, and gender. The intervention and control groups will be compared in relation to pre, post, and follow-up changes in depression, sense of belonging, social support, and mood using a mixed model between groups multivariate analysis of variance conducted using SPSS v20.

### Sample Size and power calculations

GPower [32] analysis indicates that to detect medium effect size with four outcome variables (*f* (V) test = .25) with power of at least .80 and an alpha level of .05, a sample size of 79 participants per condition is sufficient, assuming that these participants complete all questionnaires and complete the intervention program. As his study aims to assess the feasibility of playing Wii bowling in residential age care, a total sample size of 158 may not be able to be obtained. If this occurs, the current study will be marked as a pilot study.

### Ethical approval and trial registration

Ethical approval for this study was granted by Federation University Australia in May 2013. The trial has been registered with the Australian New Zealand Clinical Trial Registry since 30 April 2014: ACTRN12614000445673.

## Discussion

This research aims to identify if playing Wii bowling can benefit aged care facility residents by improving their overall psychological health. Older adults who reside in aged care facilities have higher level of depression [3], and lower levels of sense of belonging [9] and social support [33] than older adults who remain living in the community. If successful, this intervention may also lead to an increase in social interaction between residents. This research may benefit staff in aged care facilities by establishing an intervention for mental health issues that is fun, economical, and easy to use.

The proposed program enables participants in the intervention group to increase their overall sense of well-being, with the potential to increase their sense of belonging. It is expected that participation in the intervention program will also enable residents to learn new skills and also help to facilitate shared relationships within the facility. All aged care facilities involved will also be provided with a Wii console, which will remain in the facility at the end of the initial 6 week research program. The control group and intervention group will have access to a Wii console at the end of the week 6. Both groups will be shown how to play the Wii independently, and staff will be provided with training. The results will improve our understanding of sense of belonging, mood, depression, and social support in the aged care facility residents, and potentially provide a cost-effective and scalable method of improving mental health in this population.

Potential limitations have been identified. Participation in the Wii intervention may not help to alleviate overall loneliness and depression in older adults. Harper [5] has found that the aged care environment does not promote well-being or the formation of meaningful friendships. Participation in a group activity may be enjoyable for the residents involved, but the activity may not alleviate overall symptoms of depression in aged care residents. Residents are also not selected based on depression symptoms, so a floor effect may occur. To address this potential limitation, qualitative data collected through verbatim written transcripts and the post-intervention evaluation questionnaires will be used to ascertain if there are any changes in resident’s behaviour throughout the intervention period that may not be captured through the quantitative measures.

A second potential limitation is that the intervention will be compared to a control group in a separate aged care facilities. This is necessary to ensure adequate numbers in each group. Residents in the second aged care facility may have access to different services or activities already integrated into the aged care facility. Also, residents across the facilities may vary significantly on demographic variables. To minimise the potential impact of this aged care facilities from similar socio-economic areas will be selected and any differences will be statistically controlled for in the analyses.

The study is also limited by the use of self-report measures with older adults. Factors, such as acute illness, changes in physical functioning, or cognitive impairment can influence the accuracy of self-report measures in hospitalised older adults [34]. A number of residents may exhibit low level cognitive impairment as assessed by the SLUMS exam. These residents may have trouble comprehending some of the questions, resulting in inaccurate results on the self-report measure of depression, social support, and sense of belonging.

## Conclusion

Aged care residents are at a great risk of experiencing poor mental health outcomes when compared to older adults living in the community. Participation in a group activity playing Wii bowling may lead to a decrease in the levels of depression experienced, as well as higher levels of social support and sense of belonging, and increased meaningful social interaction. This intervention would be a cost-effective and easily implemented intervention if found effective.

## References

[1] World Health Organisation (2002). Active ageing: A policy framework. In A contribution of the World Health Organization to the Second United Nations World Assembly on Ageing. Madrid, Spain; April, 2002; 1-59.

[2] AIHW (2012). Residential aged care in Australia 2010–11: a statistical overview. [aged care statistics series no. 36. Cat. no. AGE 68.

[3] Snowdon J, Fleming R (2008). Recognising depression in residential facilities: an Australian challenge. Int J Geriatr Psychiatry.

[4] Pinquart M, Sörensen S (2003). Risk factors for loneliness in adulthood and old age-a meta-analysis. Adv Psychol Res Vol 19.

[5] Harper G (2002). Daily life in a nursing home: Has it changed in 25 years?. J Aging Stud.

[6] Cacioppo JT, Hughes ME, Waite LJ, Hawkley LC, Thisted RA (2006). Loneliness as a specific risk factor for depressive symptoms: cross-sectional and longitudinal analyses. Psychol Aging.

[7] Tiikkainen P, Heikkinen R (2005). Associations between loneliness, depressive symptoms and perceived togetherness in older people. Aging Ment Health.

[8] Knight T, Mellor D (2007). Social inclusion of older adults in care: is it just a question of providing activities?. Int J Qual Stud Health Well-Being.

[9] McLaren S, Turner J, Gomez R, McLachlan AJ, Gibbs PM (2013). Housing type and depressive symptoms among older adults: a test of sense of belonging as a mediating and moderating variable. Aging Ment Health.

[10] McLaren S, Gomez R, Bailey M, Van Der Horst RK (2007). The association of depression and sense of belonging with suicidal ideation among older adults: Applicability of resiliency models. Suicide Life Threat Behav.

[11] Wiemeyer J, Kliem A (2012). Serious games in prevention and rehabilitation—a new panacea for elderly people?. Eur Rev Aging Phys Act.

[12] Bleakley CM, Charles D, Porter-Armstrong A, McNeill MDJ, McDonough SM, McCormack B (2013). Gaming for health a systematic review of the physical and cognitive effects of interactive computer games in older adults. J Appl Gerontol.

[13] McGuire FA (1984). Improving the quality of life for residents of long term care facilities through video games. Act Adapt Aging.

[14] Keogh J, Power N, Wooller L, Lucas P, Whatman C (2012). Can the Nintendo WII (TM) sports game system be effectively utilized in the nursing home environment?. J Community Inform.

[15] Franco JR, Jacobs K, Inzerillo C, Kluzik J (2012). The effect of the Nintendo Wii Fit and exercise in improving balance and quality of life in community dwelling elders. Technol Health Care.

[16] Lawrence E, Sax C, Navarro KF, Qiao M (2010). Interactive games to improve quality of life for the elderly: towards integration into a WSN monitoring system. Second Int Conference on eHealth, Telemedicine, Soc Med.

[17] Cyarto E, Kuys SS, Henwood TR, Blackberry I. Can Wii™ work it out?. Telecommun J Aust 2011;61:1-12.

[18] Marston HR (2013). Digital gaming perspectives of older adults: content vs. Interaction. Educ Gerontol.

[19] Rosenberg D, Depp CA, Vahia IV, Reichstadt J, Palmer BW, Kerr J (2010). Exergames for subsyndromal depression in older adults: a pilot study of a novel intervention. Am J Geriatr Psychiatry.

[20] Kahlbaugh PE, Sperandio AJ, Carlson AL, Hauselt J (2011). Effects of playing Wii on well-being in the wlderly: physical activity, loneliness, and mood. Act Adapt Aging.

[21] Keogh JWL, Power N, Wooller L, Lucas P, Whatman C (2014). Physical and psychosocial function in residential aged-care elders: effect of Nintendo Wii sports games. J Aging Phys Act.

[22] Koay JL, Ng J, Wong GL (2009). Nintendo Wii as an intervention: improving the well-being of elderly in long-term care facilities.

[23] Cruz-Oliver DM, Malmstrom TK, Allen CM, Tumosa N, Morley JE (2012). The veterans affairs Saint Louis university mental status exam (SLUMS exam) and the mini-mental status exam as predictors of mortality and institutionalization. J Nutr Health Aging.

[24] Feliciano L, Horning SM, Klebe KJ, Anderson SL, Cornwell RE, Davis HP (2012). Utility of the SLUMS as a cognitive screening tool among a nonveteran sample of older adults. Am J Geriatr Psychiatry.

[25] Lesher EL, Berryhill JS (1994). Validation of the geriatric depression scale-short form among inpatients. J Clin Psychol.

[26] Yesavage JA, Sheikh JI (1986). Geriatric Depression Scale (GDS). Clin Gerontol.

[27] Cutrona CE, Russell DW, Jones WH, Perlman D (1987). The provisions of social relationships and adaptation to stress. Advances in personal relationships. Volume 1.

[28] Hagerty BM, Patusky KL (1995). Developing a measure of sense of belonging. Nurs Res.

[29] Morris S. Ageing under the rainbow: The interrelations between age, sense of belonging, and mental health among Australian gay men. Ballarat, Australia: University of Ballarat; 2010.

[30] Heikkinen R-L, Kauppinen M (2004). Depressive symptoms in late life: A 10-year follow-up. Arch Gerontol Geriatr.

[31] Bagley H, Cordingley L, Burns A, Mozley CG, Sutcliffe C, Challis D (2000). Recognition of depression by staff in nursing and residential homes. J Clin Nurs.

[32] Faul F, Erdfelder E, Lang A-G, Buchner A (2007). G*Power 3: A flexible statistical power analysis program for the social, behavioral, and biomedical sciences. Behav Res Methods.

[33] Park NS, Zimmerman S, Kinslow K, Shin HJ, Roff LL (2012). Social Engagement in sssisted living and implications for practice. J Appl Gerontol.

[34] Sager MA, Dunham NC, Schwantes A, Mecum L, Halverson K, Harlowe D (1992). Measurement of activities of daily living in hospitalized elderly: a comparison of self-report and performance-based methods. J Am Geriatr Soc.

